# The Causation of Carcinoma

**Published:** 1905-02-04

**Authors:** 


### THE CAUSATION OF CARCINOMA.

If perseverance and determination have their usual reward the mystery of carcinoma should surely be approaching its term, for science is immune to the heart-sickness of deferred hope. So much has been said and written by the partisans of the cellular and parasitic theories respectively, that it is little wonder if plain men assume an impenetrable scepticism at sight of the above heading, but none the less the subject is of such paramount importance that no one can afford to neglect the main steps of current research in the matter.

The latest contribution comes from Dr. Ford Robertson and Mr. Henry Wade,[1] disciples of the parasitic school, and brings the record of their investigations up to date. The incriminated parasite is one allied to the organism causing on the roots of turnips the tumours known in this country as "finger-and-toe disease," that is, the "Plasmodiophora brassicae." This organism, gaining entrance by the root-hairs of the plant, enters the cells and sets up in the course of its life-cycle the morbid cellular proliferation which results in the tumour. This life-cycle the authors have traced with completeness in the plant, and the cycle may be roughly compared to that of the malarial parasite, though in this case no immediate host appears to be necessary. They claim to have histologically identified representatives of every phase of this life-cycle in mammary and intestinal carcinoma, but the similarity borne by tissues in varying stages of degeneration to the organism as found in plants, renders this part of their evidence inconclusive, as they themselves admit.

It is in the cultivation of the alleged parasite that the research under notice becomes most convincing. By inoculating agar-tubes with portions of growths recently excised, they have achieved a growth in (not as usual on) the medium, of an organism representing a considerable number of the phases observed both in the turnip and in the carcinomata examined. Moreover, control experiments carried out under precisely similar conditions, but with fragments of benign instead of malignant tumours, have been uniformly negative.

We have grown too cautious to be enthusiastic over reported discoveries in this particular field, but it must be admitted that this communication is of the greatest interest. If the parasitic theory be ever established it may well appear that the numerous organisms from time to time identified with carcinoma are but phases in the life-cycle of a specific parasite. The crucial test, namely, reproduction of the disease in animals by means of this body, appears yet to be lacking. We may await the result in an attitude of sympathy tinged perhaps with a not unnatural scepticism.

[1] Lancet, Jan. 28, 1905.

